# [^18^F]FE‐PE2I PET is a diagnostic tool in dementia with Lewy bodies

**DOI:** 10.1002/pcn5.70123

**Published:** 2025-06-02

**Authors:** Andrea Sturchio, Jonas E. Svensson, Mikael Tiger, Anton Forsberg Morén, Andrea Varrone, Per Svenningsson, Yoshiro Okubo, Amane Tateno

**Affiliations:** ^1^ Department of Clinical Neuroscience, Neuro Svenningsson Karolinska Institute Stockholm Sweden; ^2^ Department of Neurology James J. and Joan A. Gardner Family Center for Parkinson's Disease and Movement Disorders Cincinnati Ohio USA; ^3^ Department of Clinical Neuroscience Centre for Psychiatry Research, Karolinska Institutet & Stockholm Health Care Services, Region Stockholm Stockholm Sweden; ^4^ Neurobiology Research Unit, Copenhagen University Hospital Copenhagen Denmark; ^5^ Department of Neuropsychiatry Nippon Medical School Tokyo Japan

**Keywords:** [18F]FE‐PE2I, dementia with Lewy bodies, dopamine transporter (DAT) imaging, neurodegeneration, neuroimaging

## Abstract

**Aim:**

Dementia with Lewy bodies (DLB) is characterized by motor and non‐motor symptoms. The degeneration of the dopaminergic pathway is a hallmark of DLB; for this reason, we aimed to study a recent dopamine transporter (DAT) positron emission tomography (PET) radioligand as a diagnostic tool for DLB.

**Methods:**

In this study, we used DAT–PET with the radioligand [^18^F]FE‐PE2I to distinguish DLB subjects from healthy controls (HCs). We also aimed to analyze how DAT binding correlated with clinical features, amyloid load, measured by PET, and cardiac metaiodobenzylguanidine scintigraphy (MIBG).

**Results:**

Binding potential (*BP*_ND_) values of [^18^F]FE‐PE2I were higher in HCs versus DLB in striatum (1.82 ± 0.34 vs. 1.15 ± 0.34; *p* < 0.001; 95% Confidence Interval [CI]: 0.40–0.96), putamen (2.2 ± 0.36 vs. 1.41 ± 0.51; *p* < 0.001; 95% CI: 0.39–1.17), caudate (1.38 ± 0.30 vs. 0.88 ± 0.20; *p* < 0.001; 95% CI: 0.28–0.70), and substantia nigra (0.49 ± 0.091 vs. 0.42 ± 0.084; *p* = 0.0437; 95% CI: 0.003 to 0.14). After adjusting for age, substantia nigra did not differ between DLB and HCs (*p*: 0.46; 95% CI: −0.049 to 0.11); however, *BP*_ND_ values between DLB and HC in striatum (*p*: <0.001; 95% CI: 0.25–0.85), putamen (*p*: 0.0012; 95% CI: 0.31–1.13), and caudate (*p*: 0.0027; 95% CI: 0.13–0.55) were still significant. Striatum was the best area to correctly classify DLB subjects versus HC compared to the putamen, caudate, and substantia nigra (area under the curve = 0.95, 0.90, 0.93, and 0.73, respectively; 95 CI: 0.87–1.00, 0.79–1.00, 0.84–1.00, 0.55–0.92, respectively). Subjects with altered MIBG showed lower *BP*_ND_ compared to subjects with normal MIBG in the putamen.

**Conclusion:**

Our study showed that [^18^F]FE‐PE2I PET represents a potential diagnostic tool with high accuracy in discriminating DLB patients versus HC, which is valuable for clinical practice.

## Graphical abstract

Our study showed that [18 F]FE‐PE2I PET represents a potential diagnostic tool with high accuracy in discriminating DLB patients from healthy controls, which is valuable for clinical practice.

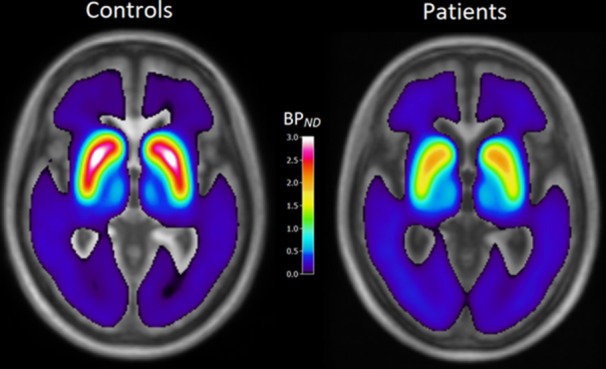

## INTRODUCTION

Dementia with Lewy bodies (DLB) is a disabling neurological condition characterized by rapidly progressing motor and non‐motor symptoms.^1^ Extrapyramidal motor signs, such as parkinsonism, rapid eye movement sleep behavior disorder, fluctuating cognitive impairment, and visual hallucination are the core symptoms of the disease.^1^, ^2^ DLB is the second most common type of dementia after Alzheimer's disease (AD).^3^ Clinically, the presentation of DLB can mimic AD and Parkinsonian syndromes (PS), making the diagnostic process challenging.^4^, ^5^ Moreover, more than 50% of DLB subjects present AD co‐pathology^6^ and the presence of AD co‐pathology is associated with worse clinical DLB outcome.^7^ There is a large unmet need for diagnostic biomarkers with high sensitivity for earlier diagnosis of DLB and better monitoring of disease progression.

Degeneration of the dopaminergic nigrostriatal pathway is a central feature in DLB patients.^8^ Dopamine transporter (DAT) imaging is a valuable diagnostic tool to detect abnormalities in the integrity of the dopaminergic system in DLB.^9^ Therefore, this diagnostic approach represents an important support that may help the clinician in the differential diagnosis of neurodegenerative disorders with versus without dopaminergic deficiency, more specifically DLB versus AD.^10^, ^11^

Currently, the majority of DAT imaging data both in clinical practice and in research are based on [123I]FP‐CIT ([123I]‐ioflupane), a single‐photon emission computed tomography (SPECT)‐based DAT tracer.^4^, ^5^ Due to the limited spatial resolution of DAT SPECT imaging, there is a need for improved imaging techniques for support in clinical diagnosis. This can be achieved by using positron emission tomography (PET) and the DAT radioligand [^18^F]‐(E)‐N‐(3‐iodoprop‐2‐enyl)‐2β‐carbofluoroethoxy‐3β‐ (4′‐methyl‐phenyl) nortropane ([^18^F]FE‐PE2I). Another potential advantage of [^18^F]FE‐PE2I is the higher affinity for DAT over the serotonin transporter, allowing [^18^F]FE‐PE2I to evaluate extra‐striatal regions, such as the substantia nigra (SN),^12^ where the DAT density is lower.^13^ In addition, the higher resolution of PET enables quantification of DAT binding in small regions like the SN.^14^, ^15^ Compared to [123I]FP‐CIT, [^18^F]FE‐PE2I PET imaging provides additional benefits with better management of the patients, including reduced imaging time due to faster kinetics of the radioligand as well as no need to protect the thyroid, reducing radiation burden.^16^

[^18^F]FE‐PE2I imaging has already shown high specificity and sensitivity in discriminating Parkinson´s disease (PD), the most common PS, from controls; moreover, [^18^F]FE‐PE2I correlates with PD clinical features.^4^ However, there is a lack of data assessing how well [^18^F]FE‐PE2I PET can separate healthy individuals and DLB cases.

Given this background, the primary aims of the study were to evaluate the difference in binding potential (*BP*_ND_) values and the area under the curve (AUC) of [^18^F]FE‐PE2I in classifying subjects with DLB versus healthy controls (HCs), analyzing different brain regions, including the SN. In addition, as exploratory aims, we examined: (a) the correlation between [^18^F]FE‐PE2I binding with motor/non‐motor symptoms and amyloid load measured using ^18^F‐florbetapir PET, to better understand the correlation with AD co‐pathology; and (b) the correlation between [^18^F]FE‐PE2I binding and cardiac metaiodobenzylguanidine scintigraphy (MIBG). The last aim was investigated since MIBG is able to detect impaired cardiac postganglionic sympathetic innervation,^17^ which is more common in DLB compared to other forms of dementia or PS.^18^, ^19^

## METHODS

The study was conducted in accordance with the ethical principles based on the Declaration of Helsinki and in compliance with the Clinical Research Law of Japan, the enforcement regulations of the Law, and other relevant notices.

### Subjects

Fifteen and 14 consecutive HC and DLB subjects, respectively, were recruited. DLB diagnosis was done following the 2017 McKeith criteria.^20^ Progressive supranuclear palsy (PSP) was ruled out by excluding typical findings of PSP: atrophy of the midbrain tegmentum; atrophy of the superior cerebellar peduncle; enlargement of the third ventricle; and atrophy of the frontal, temporal, and parietal lobes.

Inclusion criteria for HCs have been previously described.^21^ All DLB patients were outpatients or inpatients at the Department of Neuropsychiatry, Nippon Medical School Hospital. All participants signed an informed consent approved by the Institutional Review Board at Nippon Medical School Hospital.

### Clinical assessment

#### Clinical scales

The following scales were collected: (a) Alzheimer's Disease Assessment Scale –cognitive component Japanese version (ADAS‐Jcog),^22^ (b) Mini–Mental State Examination,^23^ and (c) the Japanese version of the Geriatric Depression Scale (GDS).^24^

#### Genetic data

Apolipoprotein E (*APOE*) status was collected for all the DLB subjects.

#### Other clinical data

The presence or absence of the following symptoms was also collected: (a) fluctuating cognition, (b) recurrent visual hallucinations, (c) parkinsonism, (d) REM sleep behavior disorder, (e) severe sensitivity to neuroleptics, (f) systematized delusions, and (g) depression.

### Imaging data

PET examinations were performed with an Eminence SET‐3000GCT‐X (Shimadzu Corp.) with an image resolution of 3.49 mm full‐width half‐maximum (FWHM) at 1 cm off center and 3.82–5.14 mm FWHM 10 cm off center.^25^ An inflatable head‐fixation device was used during the scans. Attenuation was corrected with a 10‐min 137Cs transmission examination. [^18^F]FE‐PE2I was synthesized from its precursor, tosylethyl‐PE2I, as described previously^26^ and injected intravenously as a bolus. Emission data were collected during 60 min (20 s × 9, 1 min × 5, 2 min × 4, 4 min × 11). Injected radioactivity was 176.7–190.4 MBq (average ± SD: 183.2 ± 3.5). Specific radioactivity was 268.7–1882 GBq/μmol (average ± SD: 622.7 ± 520.7). Image analysis was blinded to clinical diagnosis.

#### Regions of interest

FreeSurfer (version 6.0, http://surfer.nmr.mgh.harvard.edu/)^27^ was used to delineate brain regions on the T1‐weighted MRIs of all subjects. We chose to quantify the binding in regions with relevance to DLB and with acceptable signal‐to‐noise ratio. Delineation of SN was performed using the Montreal Neurological Institute (MNI) template‐based delineation of nigrostriatal tracts. The FreeSurfer‐generated cerebellar mask used as a reference region was trimmed using an automated method developed in‐house, described previously.^28^ Substantia nigra, putamen, and the caudate were analyzed individually (Figure S1). Additionally, the putamen, the caudate, and the nucleus accumbens were combined into a composite “striatum” region of interest (ROI).

#### MRI and PET image preprocessing and quantification

Dynamic PET images were corrected for head motion using a between‐frame‐correction algorithm implemented in SPM12 (Wellcome Department of Cognitive Neurology, University College). Frames were realigned to the first 4‐min frame (16–20 min of the PET examination). For each individual, the T1‐weighted MR‐image was co‐registered to a time‐weighted summated PET image, yielding a co‐registration matrix. The co‐registration matrix was then used to project ROIs on the realigned dynamic PET image to derive regional time–activity curves. From the time–activity curves, *BP*_ND_ was calculated for each ROI using the noninvasive Logan plot fitted with multilinear regression^29^; t* was set to 32 min, corresponding to seven frames. Cerebellar gray matter was used as a reference region due to its negligible concentration of DAT.^30^ The reference region efflux rate constant (*k*2') was derived using the simplified reference tissue model^31^ in striatal regions. To calculate *BP*_ND_ for the SN masks, parametric images were generated using the 3D stationary wavelet‐aided parametric imaging procedure, where the noninvasive Logan plot, fitted with multilinear regression, is applied on time activity curves from individual voxels.^32^ This method has been shown to effectively reduce noise in small brain regions.^33^ For each individual, the SN mask was applied to the parametric image to obtain the binding estimate in this small brain region. For visualizations, the parametric images were registered to MNI‐space^34^ and averaged across patients and controls, respectively.

#### Amyloid‐PET imaging

Amyloid‐PET imaging was performed using an Eminence SET‐3000GCT/X (Shimadzu Corp.) system and [^18^F]florbetapir, with quantitative analysis performed as previously described.^35^ In short, the binding estimates for six brain areas (medial orbital frontal, temporal, anterior cingulate, posterior cingulate, parietal lobe, and precuneus) were averaged and used as an index of amyloid load. Data were reported as standardized uptake values (SUVR).

#### Cardiac MIBG

Cardiac MIBG was performed using Philips Bright View (matrix 256 × 256, imagine tie 10 min). Then, 118 MBq of [^123^I] metaiodobenzylguanidine (^123^I‐MIBG) were injected and the heart‐to‐mediastinal ratio (HMR) was calculated. HMR values below 2.2 were considered abnormal. Both early and late HMR images are used for evaluation. We then consider sympathetic neuropathy when the HMR ratio and washout ratio (WR) meet all the following: (1) HMR of early phase images is <2.2; (2) HMR of the late phase images is less than the HMR of the early phase images; (3) WR is >30%.^36^

The collimator used was a MEGP (medium energy general‐purpose type). The MIBG washout period was approximately 3 h and 45 min, with the early image taken 15 min after I‐MIBG administration and the late image taken approximately 4 h after administration. Data were analyzed using SMART MIBG software.

### Aims and statistical analysis

With the present study, the primary aims were to evaluate: (a) the difference in the *BP*_ND_ estimated using Logan graphical analysis with the cerebellum as a reference region; and (b) the area under the curve of [^18^F]FE‐PE2I in classifying DLB subjects versus HC in different brain regions, such as the entire striatum (putamen, caudate, and nucleus accumbens), putamen, caudate, and SN. Exploratory aims were to investigate whether [^18^F]FE‐PE2I binding correlates with motor/nonmotor symptoms, amyloid load, and cardiac MIBG.

Tests for normal distribution were carried out using the Shapiro–Wilk test. Comparison between the two groups was performed using parametric *t*‐test/Mann–Whitney *U*/Fisher's exact test according to data distribution. Adjusted analyses were performed using ANCOVA and data were reported as *p*‐value and confidence interval (CI). The receiver operating characteristic curve (ROC) was calculated and the total AUC was reported. Correlations between [^18^F]FE‐P2I binding and clinical features were assessed using Spearman or Pearson according to data distribution. The two‐sided *p*‐value significance was set at 0.05. All the statistical analyses were carried out using GraphPad Prism (Version 9.5.1; GraphPad Software Inc.).

## RESULTS

A total of 15 HC and 18 DLB subjects were recruited; among the recruited DLB subjects, a total of 14 subjects met the inclusion criteria, with no exclusion criteria, and were included in the study. Clinical and demographic characteristics are shown in Table 1. DLB subjects were significantly older than HC (76.7 ± 5.4 vs. 69.3 ± 5.8; *p*: 0.0016; 95% CI: −11.69 to −3.07; Table 1) and this difference was accounted for in the statistical analyses as described later. However, no significant difference was present regarding gender distribution (*p*: 0.1431; 95% CI: Table 1). Clinical data were available for all the DLB subjects, except for ADAS‐Cog, which was available for 12 out of 14 DLB subjects.

**Table 1:** Clinicodemographic characteristics of the study population.

	HCs (15)	DLB (14)	*p* value
Age	69.3 (5.9)	76.7 (5.4)	0.0016
Female (%)	5 (33.3%)	9 (64%)	NS
ADAS‐Cog^a^	—	15.3 (10.2)	—
MMSE	—	22.4 (5.4)	—
GDS	—	4.8 (3.2)	—
APOE	—		—
E3/3		6 (42.9%)	
E3/2		4 (28.6%)	
E4/3		3 (21.4%)	
E3/5		1 (7.1%)	
Fluctuating cognition	—	8 (57.1%)	—
Recurrent visual hallucinations	—	12 (85.7%)	—
Parkinsonism	—	11 (78.6%)	—
REM sleep behavior disorder	—	7 (50%)	—
Severe sensitivity to neuroleptics	—	6 (42.9%)	—
Systematized delusions	—	7 (50%)	—
Depression	—	10 (71.4%)	—
Amyloid‐PET (SUVR)		1.2 (0.2)	
Decreased uptake of MIBG	—	5 (35.7%)	—
Other medication		SSRI: 4 (28.6%)	—
		NaSSA: 1 (7.1%)	
		AChEIs: 8 (57.1%)	
		SARIs: 1 (7.1%)	
		AAP: 5 (35.7%)	
		Mood stabilizer: 1 (7.1%)	

*Note*: Data are expressed as mean ± standard deviation (SD) or frequency (%).

Abbreviations: AAP, atypical antipsychotics; AChEIs, acetylcholinesterase inhibitors; ADAS‐Cog, Alzheimer's Disease Assessment Scale–Cognitive subscale; APOE, apolipoprotein E; DLB, dementia with Lewy bodies; GDS, Geriatric Depression Scale; HCs, healthy controls; MIBG, metaiodobenzylguanidine scintigraphy; MMSE, Mini–Mental State Examination; NaSSA, noradrenergic and specific serotonergic antidepressants; NS, not significant; PET, positron emission tomography; SARIs, serotonin receptor antagonists and reuptake inhibitors; SSRI, selective serotonin reuptake inhibitors; SUVR, standardized uptake value.

^a^ Available for 12 subjects.

### [^18^F]FE‐PE2I *BP*_ND_ comparison between DLB versus HC in different brain areas

**Figure 1:**
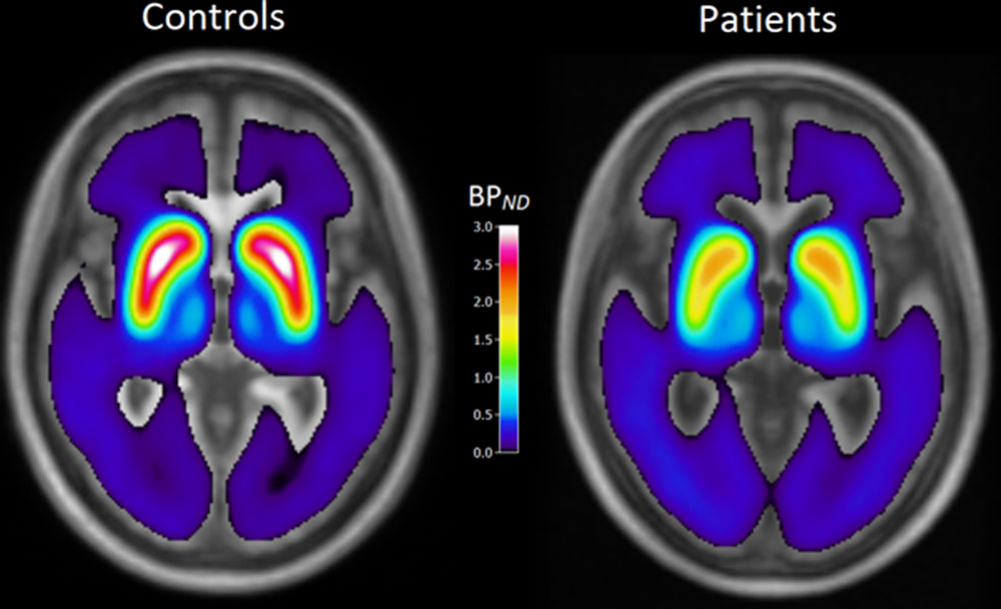
Mean wavelet‐aided parametric images of [^18^F]FE‐PE2I *BP*_ND_ in healthy control subjects (left) and subjects with dementia with Lewy bodies (right).

**Figure 2:**
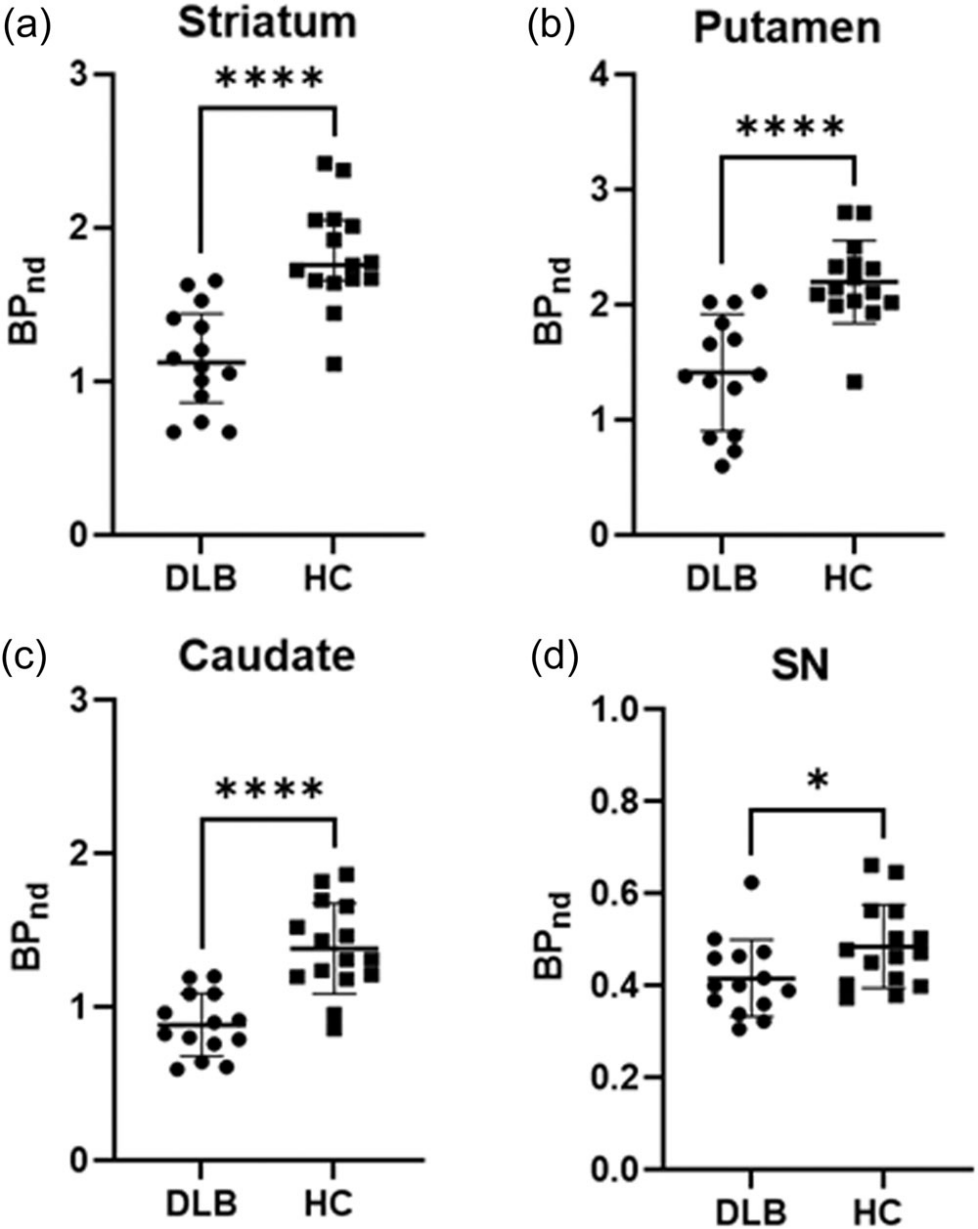
Differences between [^18^F]FE‐PE2I *BP*_ND_ values between subjects with dementia with Lewy bodies (DLB) versus healthy controls (HCs) in (a) in the striatum, (b) putamen, (c) caudate, and (d) substantia nigra. *BP*_ND_, binding potential. Data are represented as mean ± standard deviation.

**Figure 3:**
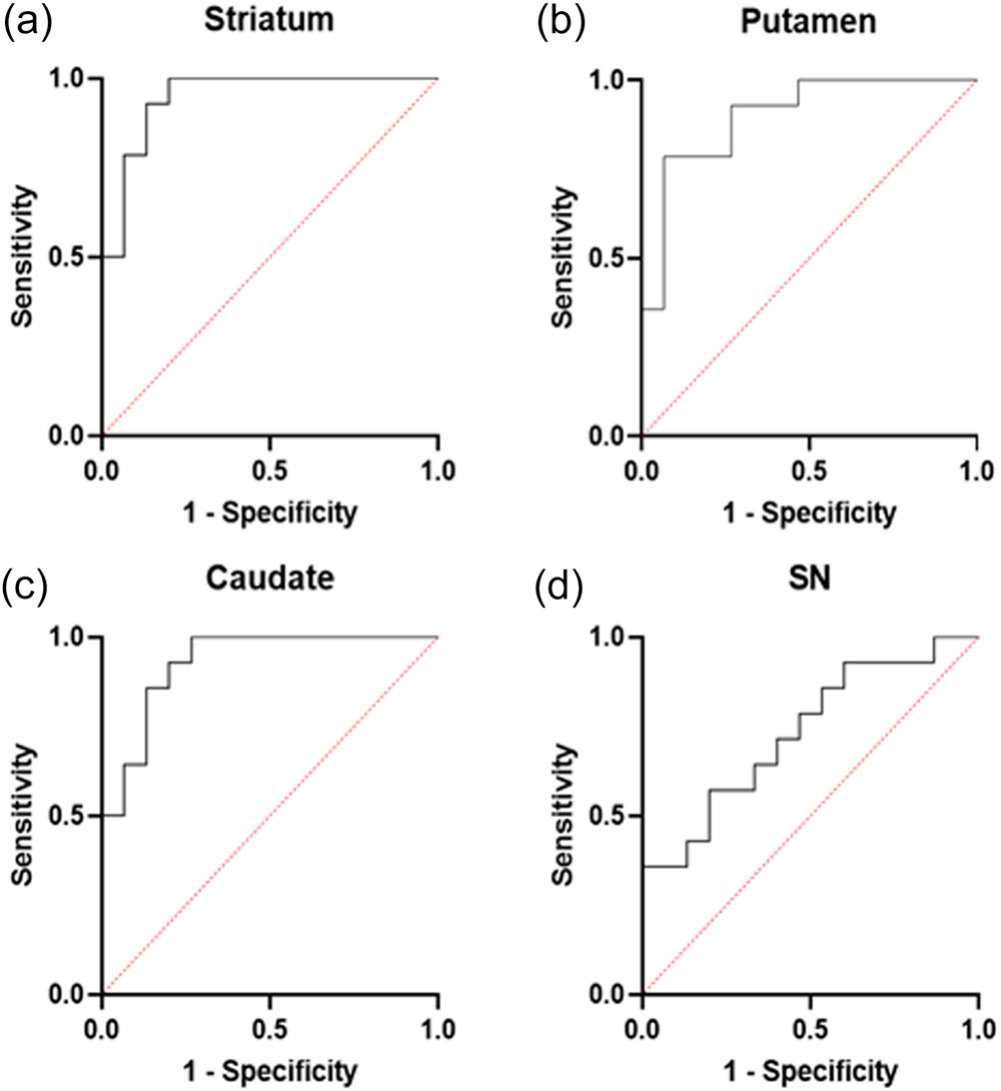
Receiver operating characteristic (ROC) curve of [^18^F]FE‐PE2I *BP*_ND_ in detecting dementia with Lewy bodies (DLB) subjects in (a) the striatum, (b) putamen, (c) caudate, and (d) substantia nigra. HC, healthy controls; *BP*_ND_, binding potential. Data are represented as mean ± standard deviation.

[^18^F]FE‐P2I *BP*_ND_ values were significantly higher in HCs versus DLB in the striatum (1.82 ± 0.34 vs. 1.15 ± 0.34; *p* < 0.001; 95% CI: 0.40–0.96), putamen (2.2 ± 0.36 vs. 1.41 ± 0.51; *p* < 0.001; 95% CI: 0.39–1.17), caudate (1.38 ± 0.30 vs. 0.88 ± 0.20; *p* < 0.001; 95% CI: 0.28–0.70), and SN (0.49 ± 0.091 vs. 0.42 ± 0.084; *p* = 0.0437; 95% CI: 0.003 to 0.14) (Figures 1 and 2). After adjusting for age, substantia nigra did not significantly differ between DLB and HC (*p*: 0.46; 95% CI: −0.049 to 0.11), whereas *BP*_ND_ values in striatum (*p*: <0.001; 95% CI: 0.25–0.85), putamen (*p*: 0.0012; 95% CI: 0.31–1.13), and caudate (*p*: 0.0027; 95% CI: 0.13–0.55) were still significantly higher in controls. Classification of DLB subjects versus HCs was done better using *BP*_ND_ values in the striatum than putamen, caudate, and SN (AUC = 0.95, 0.90, 0.93, and 0.73, 95% CI: 0.87–1.00, 0.79–1.00, 0.84–1.00, 0.55–0.92, respectively) (Figure 3).

### Correlation with amyloid scans and cardiac MIBG

**Figure 4:**
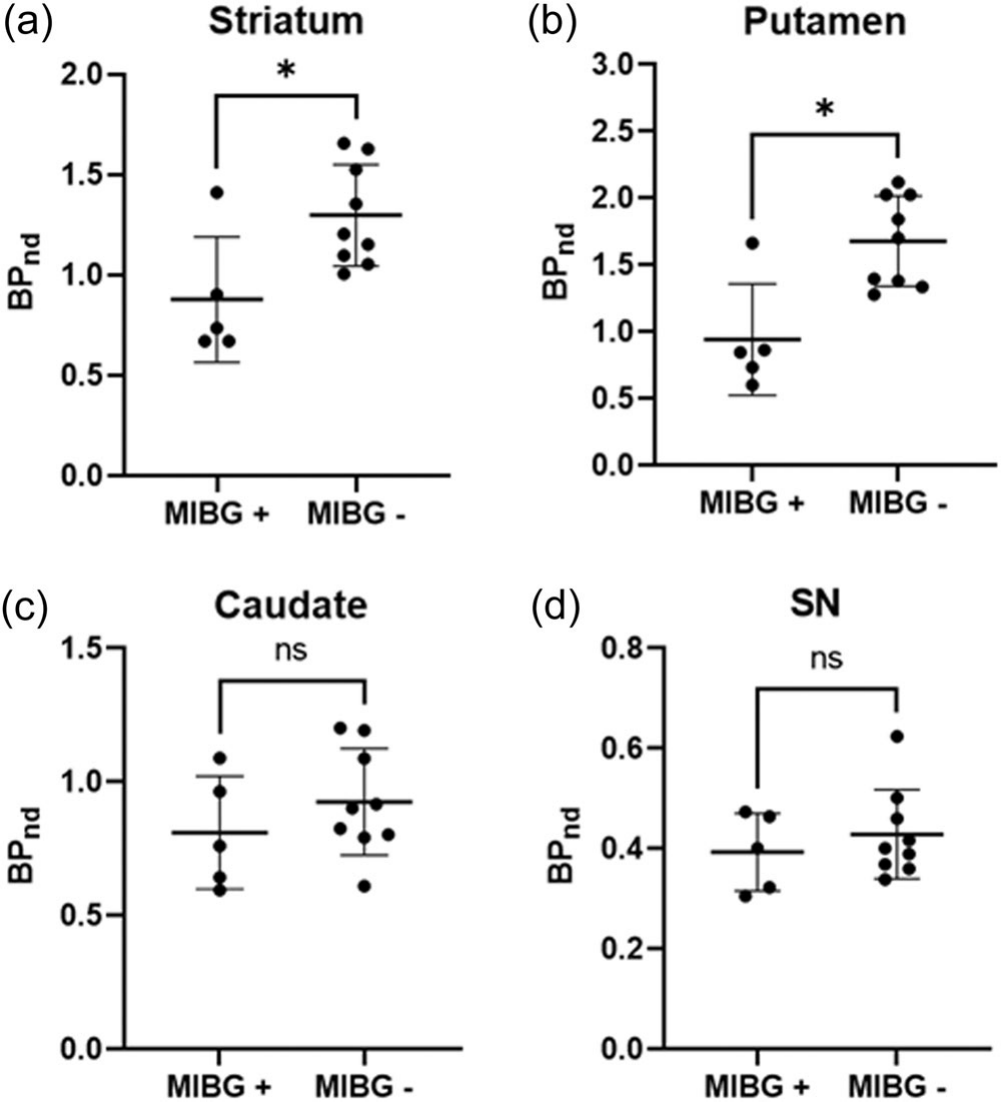
Differences between [^18^F]FE‐PE2I *BP*_ND_ values between subjects with dementia with Lewy bodies (DLB) metaiodobenzylguanidine negative (MIBG−) and MIBG positive (MIBG+) in the (a) striatum, (b) putamen, (c) caudate, and (d) substantia nigra. HC, healthy controls; *BP*_ND_, binding potential. Data are represented as mean ± standard deviation.

No significant correlations between [^18^F]FE‐PE2I *BP*_ND_ values versus amyloid SUVR were found (Tables S1–S4). MIBG scan was divided into MIBG positive (MIBG+; 5 subjects) versus MIBG negative (MIBG−; 9 subjects). MIBG+ subjects showed lower [^18^F]FE‐P2I *BP*_ND_ values compared to MIBG− in the striatum (0.88 ± 0.31 vs. 1.30 ± 0.25; *p* = 0.0290; 95% CI: 0.12–0.85) and putamen (0.94 ± 0.42 vs. 1.68 ± 0.34 *p* = 0.0120; 95% CI: 0.36–1.25), but not in the caudate (0.81 ± 0.21 vs. 0.93 ± 0.20; *p* = 0.3636; 95% CI: −0.17–0.43) or in the SN (0.39 ± 0.078 vs. 0.43 ± 0.089; *p* = 0.6064; 95% CI: −0.07–0.15) (Figure 4).

### Correlation with clinical data

No significant correlations were found between clinical data and [^18^F]FE‐PE2I *BP*_ND_ values in the striatum, caudate, putamen, and SN (Tables S1–S4).

## DISCUSSION

To the best of our knowledge, this is the first study assessing the use of [^18^F]FE‐PE2I PET in differentiating subjects affected by DLB versus HC. A previous study analyzed [^18^F]FE‐PE2I in DLB versus PD; however, this study did not assess the SN and lacks a comparison with HC.^37^ Our results support the use of [^18^F]FE‐PE2I as a diagnostic tool; in fact, we found significantly lower [^18^F]FE‐PE2I *BP*_ND_ value levels in the striatum, caudate, and putamen of DLB subjects, in line with previous data using the more widely used SPECT‐based [123I]FP‐CIT tracer.^9^ Moreover, our study showed that lower [^18^F]FE‐PE2I *BP*_ND_ values in the striatum and putamen were associated with a reduced uptake in the cardiac MIBG SPECT. However, in our analysis, [^18^F]FE‐PE2I binding did not show any correlation either with the clinical outcomes evaluated or with the amyloid burden, possibly due to the small sample size and the lack of dynamic range.

DLB is a common neurological condition; nevertheless, the diagnostic process can be challenging since it can mimic other highly prevalent neurodegenerative diseases, such as AD and PS. Early identification of these subjects can help in the clinical management of DLB patients. In addition, future clinical trials can benefit from an accurate diagnosis using an improved method compared to SPECT imaging, improving the patients' selection.^38^ Despite the loss of dopaminergic neurons being a hallmark of DLB, the presence of extrapyramidal symptoms can be mild or even absent until the last stages of the disease.^1^ Hence, the detection of a dopaminergic deficit is essential for the diagnostic process, allowing a fast and accurate diagnosis. In our analysis, [^18^F]FE‐PE2I PET was able to detect dopaminergic deficit not only in the striatum (as in previous DAT imaging studies) but also in the SN; however, the difference was not present after correcting for age. Further studies with larger sample size are needed to confirm the validity of [^18^F]FE‐PE2I PET in detecting dopaminergic deficits in SN of DLB subjects. The decrease of the dopaminergic neurons in SN is crucial for the development of the symptoms in DLB and other PSs, such as Parkinson's disease, and the possibility of analyzing the SN opens exciting new research perspectives. In particular, the striatum was the region showing the highest accuracy in discriminating DLB versus HC with an AUC of 0.95. Interestingly, we observed lower caudate *BP*_ND_ compared to putamen; this is in line with previous studies in which they showed that DAT reduction in caudate is particularly affected in DLB subjects.^39^ For instance, the dopaminergic deficit in the caudate has been associated with cognitive impairment, which is one of the typical clinical features of DLB.^40^ These data demonstrate a profound degeneration of the dopaminergic system which involves both motor and nonmotor circuitries.

On top of the primary aims of the study, we also compared [^18^F]FE‐PE2I *BP*_ND_ values with cardiac MIBG. Cardiac MIBG evaluates the degree of cardiac denervation, distinguishing between conditions affected by postganglionic (MIBG+) damage, namely DLB and PD, versus pre‐ganglionic conditions (MIBG−), such as multiple systems atrophy or progressive supranuclear palsy^18^, ^19^; moreover, MIBG could help in discriminating DLB versus AD, the latter showing normal cardiac uptake.^20^ Cardiac MIBG is an important complementary tool and it has been shown to have good accuracy in identifying subjects with DLB^16^ even in the early stage of the disease.^41^ We found that MIBG− DLB subjects had higher [^18^F]FE‐PE2I *BP*_ND_ in the striatum and putamen compared to MIBG+ DLB subjects. These results are in line with previous results conducted using [^18^F]FE‐PE2I,^31^ corroborating the validity of [^18^F]FE‐PE2I PET as a diagnostic tool for DLB. A decrease in the MIBG uptake has been associated with the presence of PS symptoms, particularly autonomic dysfunction, such as orthostatic hypotension.^42^ A larger study using specific scales is needed to evaluate the correlation between DLB symptoms and [^18^F]FE‐PE2I *BP*_ND_. We also did not find any correlation with the amyloid burden and the dopaminergic deficit, probably supporting the fact that the degenerative process in DLB has, at least in part, a separate pathophysiological mechanism.

Some limitations are present in our analysis. First, the small sample size could have masked possible correlations in the exploratory outcomes; however, the sample size was large enough to clearly detect differences in the primary outcome. Second, some clinical data lack specific scales, making it difficult to have a precise correlation with clinical features. Third, the caudate ROI is adjacent to the lateral ventricle. This will cause a spill‐out of radioactivity to a greater extent compared to the putamen, resulting in apparent lower *BP*_ND_.

## CONCLUSION

In conclusion, [^18^F]FE‐PE2I PET imaging was able to clearly discriminate DLB subjects from HCs and could represent a high‐resolution alternative to SPECT‐based imaging, potentially improving the management of these patients. The use of [^18^F]FE‐PE2I in molecular imaging of the DAT in DLB could also improve the selection of patients for future clinical trials.

## AUTHOR CONTRIBUTIONS

*Conception and design of the study*: Yoshiro Okubo and Amane Tateno. *Acquisition of the data*: Amane Tateno. *Analysis of data*: Andrea Sturchio, Anton Forsberg Morén, Jonas E. Svensson, Mikael Tiger. *First draft of the manuscript*: Andrea Sturchio, Mikael Tiger, Jonas E. Svensson. *Figures*: Andrea Sturchio, Mikael Tiger, Jonas E. Svensson. All authors read it and provided feedback.

## CONFLICT OF INTEREST STATEMENT

The authors declare no conflict of interest or other interest that might be perceived to influence the results and/or discussion reported in this paper. Andrea Sturchio is a co‐founder of REGAIN Therapeutics and is a co‐inventor of the patent “Compositions and methods for treatment and/or prophylaxis of proteinopathies.” Andrea Sturchio received payment from Baillie Gifford. Per Svenningsson has received honoraria from Sanofi Genzyme and Shire/Takeda.

### ETHICS APPROVAL STATEMENT

This study was approved by the Nippon Medical School Hospital Institutional Review Board (#221038 and #223043).

## PATIENT CONSENT STATEMENT

Written informed consent was obtained from all participants.

## CLINICAL TRIAL REGISTRATION

UMIN Clinical Trials Registry UMIN000003608/UMIN000006962.

## Supporting information

Supplementary material R1.

Supplementary Figure S1.

## Data Availability

The datasets used and/or analyzed during the current study are available from the corresponding author on reasonable request.

## References

[1] Outeiro TF , Koss DJ , Erskine D , Walker L , Kurzawa‐Akanbi M , Burn D , et al. Dementia with Lewy bodies: an update and outlook. Mol Neurodegener. 2019;14(1):5. 10.1186/s13024-019-0306-8 30665447 PMC6341685

[2] Tateno A , Nogami T , Sakayori T , Yamamoto K , Okubo Y . Depression as a prodromal symptom of neurodegenerative diseases. J Nippon Med Sch. 2023;90(2):157–164. 10.1272/jnms.JNMS.2023_90-216 37258256

[3] Sabbagh MN , Taylor A , Galasko D , Galvin JE , Goldman JG , Leverenz JB , et al. Listening session with the US Food and Drug Administration, Lewy Body Dementia Association, and an expert panel. Alzheimer's Dement (N.Y.). 2023;9(1):e12375. 10.1002/trc2.12375 36873923 PMC9983146

[4] Kerstens VS , Fazio P , Sundgren M , Halldin C , Svenningsson P , Varrone A . [^18^F]FE‐PE2I DAT correlates with Parkinson's disease duration, stage, and rigidity/bradykinesia scores: a PET radioligand validation study. EJNMMI Res. 2023;13(1):29. 10.1186/s13550-023-00974-7 37017878 PMC10076455

[5] Fazio P , Svenningsson P , Cselényi Z , Halldin C , Farde L , Varrone A . Nigrostriatal dopamine transporter availability in early Parkinson's disease. Mov Disorders. 2018;33(4):592–599. 10.1002/mds.27316 29436751

[6] Toledo JB , Abdelnour C , Weil RS , Ferreira D , Rodriguez‐Porcel F , Pilotto A , et al. Dementia with Lewy bodies: impact of co‐pathologies and implications for clinical trial design. Alzheimer's Dement. 2023;19(1):318–332. 10.1002/alz.12814 36239924 PMC9881193

[7] Tan JH , Laurell AA , Sidhom E , Rowe JB , O'Brien JT . The effect of Amyloid and Tau Co‐pathology on disease progression in Lewy body dementia: a systematic review. Parkinsonism Rel Disord. 2025;131:107255. 10.1016/j.parkreldis.2024.107255 39742695

[8] Huber M , Beyer L , Prix C , Schönecker S , Palleis C , Rauchmann BS , et al. Metabolic correlates of dopaminergic loss in dementia with Lewy bodies. Mov Disorders. 2020;35(4):595–605. 10.1002/mds.27945 31840326

[9] Jreige M , Kurian GK , Perriraz J , Potheegadoo J , Bernasconi F , Stampacchia S , et al. The diagnostic performance of functional dopaminergic scintigraphic imaging in the diagnosis of dementia with Lewy bodies: an updated systematic review. Eur J Nucl Med Mol Imaging. 2023;50(7):1988–2035. 10.1007/s00259-023-06154-y 36920494 PMC10199865

[10] O'Brien JT , Colloby S , Fenwick J , Williams ED , Firbank M , Burn D , et al. Dopamine transporter loss visualized with FP‐CIT SPECT in the differential diagnosis of dementia with Lewy bodies. Arch Neurol. 2004;61(6):919–925. 10.1001/archneur.61.6.919 15210531

[11] Walker Z , Jaros E , Walker RWH , Lee L , Costa DC , Livingston G , et al. Dementia with Lewy bodies: a comparison of clinical diagnosis, FP‐CIT single photon emission computed tomography imaging and autopsy. J Neurol Neurosurg Psychiatry. 2007;78(11):1176–1181. 10.1136/jnnp.2006.110122 17353255 PMC2117602

[12] Varrone A , Steiger C , Schou M , Takano A , Finnema SJ , Guilloteau D , et al. In vitro autoradiography and in vivo evaluation in cynomolgus monkey of [^18^F]FE‐PE2I, a new dopamine transporter PET radioligand. Synapse. 2009;63(10):871–880. 10.1002/syn.20670 19562698

[13] Marner L , Korsholm K , Anderberg L , Lonsdale MN , Jensen MR , Brødsgaard E , et al. Correction: [^18^F]FE‐PE2I PET is a feasible alternative to [123I]FP‐CIT SPECT for dopamine transporter imaging in clinically uncertain parkinsonism. EJNMMI Res. 2023;13(1):19. 10.1186/s13550-023-00970-x 36856900 PMC9978043

[14] Cuocolo A , Petretta M . PET and SPECT specialty grand challenge. When knowledge travels at the speed of light, photons take to the field. Front Nucl Med. 2021;1:671914. 10.3389/fnume.2021.671914 39355642 PMC11440946

[15] Kerstens VS , Fazio P , Sundgren M , Brumberg J , Halldin C , Svenningsson P , et al. Longitudinal DAT changes measured with [18F]FE‐PE2I PET in patients with Parkinson's disease; a validation study. NeuroImage Clin. 2023;37:103347. 10.1016/j.nicl.2023.103347 36822016 PMC9978841

[16] Jakobson Mo S , Axelsson J , Jonasson L , Larsson A , Ögren MJ , Ögren M , et al. Dopamine transporter imaging with [^18^F]FE‐PE2I PET and [123I]FP‐CIT SPECT—a clinical comparison. EJNMMI Res. 2018;8(1):100. 10.1186/s13550-018-0450-0 30443684 PMC6238014

[17] Courbon F , Brefel‐Courbon C , Thalamas C , Alibelli MJ , Berry I , Montastruc JL , et al. Cardiac MIBG scintigraphy is a sensitive tool for detecting cardiac sympathetic denervation in Parkinson's disease. Mov Disorders. 2003;18(8):890–897. 10.1002/mds.10461. 12889078

[18] Langston JW , Wiley JC , Tagliati M . Optimizing Parkinson's disease diagnosis: the role of a dual nuclear imaging algorithm. NPJ Parkinson's Dis. 2018;4:5. 10.1038/s41531-018-0041-9 29507872 PMC5824845

[19] Slaets S , Van Acker F , Versijpt J , Hauth L , Goeman J , Martin JJ , et al. Diagnostic value of MIBG cardiac scintigraphy for differential dementia diagnosis. Int J Geriatr Psychiatry. 2015;30(8):864–869. 10.1002/gps.42299 25363642 PMC4657469

[20] McKeith IG , Boeve BF , Dickson DW , Halliday G , Taylor JP , Weintraub D , et al. Diagnosis and management of dementia with Lewy bodies: Fourth consensus report of the DLB Consortium. Neurology. 2017;89(1):88–100. 10.1212/WNL.0000000000004058 28592453 PMC5496518

[21] Tateno A , Sakayori T , Kawashima Y , Higuchi M , Suhara T , Mizumura S , et al. Comparison of imaging biomarkers for Alzheimer's disease: amyloid imaging with [18F]florbetapir positron emission tomography and magnetic resonance imaging voxel‐based analysis for entorhinal cortex atrophy. Int J Geriatr Psychiatry. 2015;30(5):505–513. 10.1002/gps.4173 25043833

[22] Homma A , Fukuzawa K , Tsukada Y , Ishii T , Hasegawa K , Mohs RC . Development of a Japanese version of Alzheimer's Disease Assessment Scale (ADAS). Jpn J Geriatr Psychiatry. 1992;3:647–655, (in Japanese).

[23] Folstein MF , Folstein SE , McHugh PR . Mini‐mental state. J Psychiatr Res. 1975;12(3):189–198. 10.1016/0022-3956(75)90026-6 1202204

[24] Sugishita K , Sugishita M , Hemmi I , Asada T , Tanigawa T . A validity and reliability study of the Japanese version of the Geriatric Depression Scale 15 (GDS‐15‐J). Clin Gerontol. 2017;40(4):233–240. 10.1080/07317115.2016.1199452 28452641

[25] Tiger M , Gärde M , Tateno A , Matheson GJ , Sakayori T , Nogami T , et al. A positron emission tomography study of the serotonin1B receptor effect of electroconvulsive therapy for severe major depressive episodes. J Affect Disord. 2021;294:645–651. 10.1016/j.jad.2021.07.060 34332365

[26] Shingai Y , Tateno A , Arakawa R , Sakayori T , Kim W , Suzuki H , et al. Age‐related decline in dopamine transporter in human brain using PET with a new radioligand [^18^F]FE‐PE2I. Ann Nucl Med. 2014;28(3):220–226. 10.1007/s12149-013-0798-1 24385293

[27] Fischl B . FreeSurfer. Neuroimage. 2012;62(2):774–781. 10.1016/j.neuroimage.2012.01.021 22248573 PMC3685476

[28] Svensson JE , Schain M , Plavén‐Sigray P , Cervenka S , Tiger M , Nord M , et al. Validity and reliability of extrastriatal [11C]raclopride binding quantification in the living human brain. Neuroimage. 2019;202:116143. 10.1016/j.neuroimage.2019.116143 31473354

[29] Matheson GJ , Stenkrona P , Cselényi Z , Plavén‐Sigray P , Halldin C , Farde L , et al. Reliability of volumetric and surface‐based normalisation and smoothing techniques for PET analysis of the cortex: a test‐retest analysis using [11C]SCH‐23390. Neuroimage. 2017;155:344–353. 28419852 10.1016/j.neuroimage.2017.04.031

[30] Darcourt J , Booij J , Tatsch K , Varrone A , Vander Borght T , Kapucu ÖL , et al. EANM procedure guidelines for brain neurotransmission SPECT using (123)I‐labelled dopamine transporter ligands, version 2. Eur J Nucl Med Mol Imaging. 2010;37(2):443–450. 10.1007/s00259-009-1267-x 19838702

[31] Lammertsma AA , Hume SP . Simplified reference tissue model for PET receptor studies. Neuroimage. 1996;4(3):153–158. 10.1006/nimg.1996.0066 9345505

[32] Cselényi Z , Olsson H , Farde L , Gulyás B . Wavelet‐aided parametric mapping of cerebral dopamine D2 receptors using the high affinity PET radioligand [11C]FLB 457. Neuroimage. 2002;17(1):47–60. 10.1006/nimg.2002.1152 12482067

[33] Schain M , Tóth M , Cselényi Z , Arakawa R , Halldin C , Farde L , et al. Improved mapping and quantification of serotonin transporter availability in the human brainstem with the HRRT. Eur J Nucl Med Mol Imaging. 2013;40(2):228–237. 10.1007/s00259-012-2260-3 23076621

[34] Evans AC , Collins DL , Mills SR , Brown ED , Kelly RL , Peters TM . 3D statistical neuroanatomical models from 305 MRI volumes. 1993 IEEE Conference Record Nuclear Science Symposium and Medical Imaging Conference. 1993;3:1813–1817. 10.1109/NSSMIC.1993.373602

[35] Fleisher AS . Using positron emission tomography and florbetapir F18 to image cortical amyloid in patients with mild cognitive impairment or dementia due to Alzheimer disease. Arch Neurol. 2011;68(11):1404–1411. 10.1001/archneurol.2011.150 21747008

[36] Nakajima K , Okuda K , Matsuo S , Wakabayashi H , Kinuya S . Is ^123^I‐metaiodobenzylguanidine heart‐to‐mediastinum ratio dependent on age? From Japanese Society of Nuclear Medicine normal database. Ann Nucl Med. 2018;32(3):175–181. 10.1007/s12149-018-1231-6 29333564 PMC5852176

[37] Fedorova TD , Knudsen K , Horsager J , Hansen AK , Okkels N , Gottrup H , et al. Dopaminergic dysfunction is more symmetric in dementia with Lewy bodies compared to Parkinson's disease. J Parkinson's Dis. 2023;13(4):515–523. 10.3233/JPD-230001 37212074 PMC10357144

[38] Thomas AJ , Mahin‐Babaei F , Saidi M , Lett D , Taylor JP , Walker L , et al. Improving the identification of dementia with Lewy bodies in the context of an Alzheimer's‐type dementia. Alzheimer's Res Ther. 2018;10(1):27. 10.1186/s13195-018-0356-0 29490691 PMC5831205

[39] Iwabuchi Y , Shiga T , Kameyama M , Miyazawa R , Seki M , Ito D , et al. Striatal dopaminergic depletion pattern reflects pathological brain perfusion changes in Lewy body diseases. Mol Imaging Biol. 2022;24(6):950–958. 10.1007/s11307-022-01745-x 35701723 PMC9681681

[40] Ekman U , Eriksson J , Forsgren L , Mo SJ , Riklund K , Nyberg L . Functional brain activity and presynaptic dopamine uptake in patients with Parkinson's disease and mild cognitive impairment: a cross‐sectional study. Lancet. Neurol. 2012;11(8):679–687. 10.1016/S1474-4422(12)70138-2 22742929

[41] Roberts G , Durcan R , Donaghy PC , Lawley S , Ciafone J , Hamilton CA , et al. Accuracy of cardiac innervation scintigraphy for mild cognitive impairment with Lewy bodies. Neurology. 2021; 96(23):e2801–e2811. 10.1212/WNL.0000000000012060 33883238 PMC8205462

[42] Kobayashi S , Tateno M , Morii H , Utsumi K , Saito T . Decreased cardiac MIBG uptake, its correlation with clinical symptoms in dementia with Lewy bodies. Psychiatry Res. 2009;174(1):76–80. 10.1016/j.pscychresns.2009.02.006 19766460

