# Supplementary material for: [18F]FE‐PE2I PET is a diagnostic tool in dementia with Lewy bodies
Source: PCN Rep. 2025 Jun 2;4(2):e70123. doi: 10.1002/pcn5.70123 (PMC12128163; doi:10.1002/pcn5.70123)
Supplement: Supplementary file 1 — Supplementary material R1. [file PCN5-4-e70123-s002.docx]

**Supplementary material 1:** **Correlation between *BP*_ND_ in Substantia Nigra, clinical scales, and amyloid SUVR**

|  | Coefficient | p-value |
| --- | --- | --- |
| ADAS-cog | r: -0,2316 | ns |
| MMSE | r: 0,2190 | ns |
| GDS | r: -0,07752 | ns |
| Amyloid SUVR | r: -0,5341 | ns |

**Supplementary material 2:** **Correlation between *BP*_ND_ in striatum, clinical scales, and amyloid SUVR**

|  | Coefficient | p-value |
| --- | --- | --- |
| ADAS-cog | r: 0,1544 | ns |
| MMSE | r: -0,05089 | ns |
| GDS | r: -0,3544 | ns |
| Amyloid SUVR | r: -0,05934 | ns |

**Supplementary material 3:** **Correlation between *BP*_ND_ in caudate, clinical scales, and amyloid SUVR**

|  | Coefficient | p-value |
| --- | --- | --- |
| ADAS-cog | r: 0,1895 | ns |
| MMSE | r: -0,2522 | ns |
| GDS | r: -0,3300 | ns |
| Amyloid SUVR | r: -0,1033 | ns |

**Supplementary material 4:** **Correlation between *BP*_ND_ in putamen, clinical scales, and amyloid SUVR**

|  | Coefficient | p-value |
| --- | --- | --- |
| ADAS-cog | r: 0,2211 | ns |
| MMSE | r: -0,09513 | ns |
| GDS | r: -0,3300 | ns |
| Amyloid SUVR | r: 0,04176 | ns |
