# Supplementary figures and images for: [18F]FE‐PE2I PET is a diagnostic tool in dementia with Lewy bodies
Source: PCN Rep. 2025 Jun 2;4(2):e70123. doi: 10.1002/pcn5.70123 (PMC12128163; doi:10.1002/pcn5.70123)

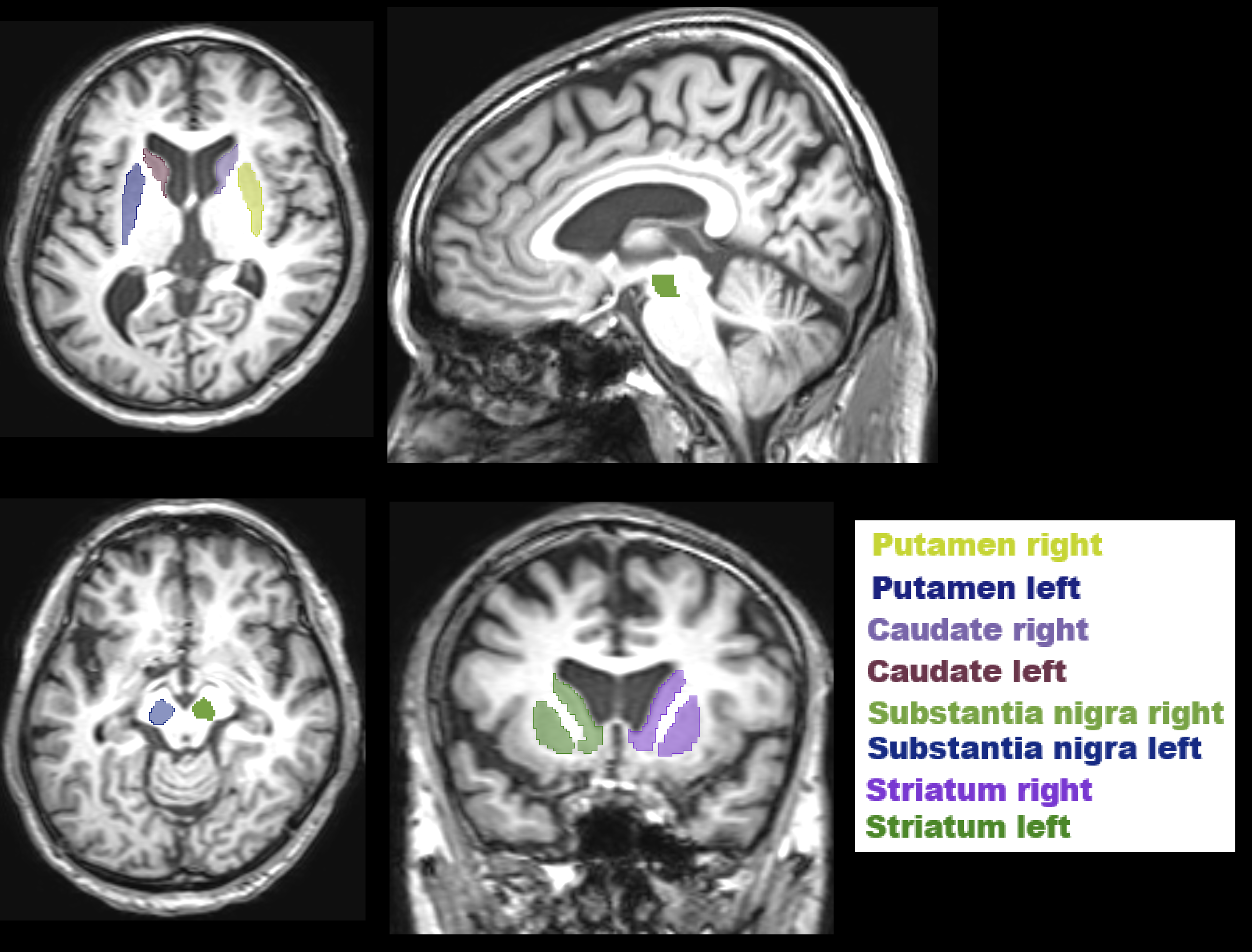

Supplement: Supplementary file 2 — Supplementary Figure S1. [file PCN5-4-e70123-s001.png]
